# Comparative network analysis reveals the dynamics of organic acid diversity during fruit ripening in peach (*Prunus persica* L. Batsch)

**DOI:** 10.1186/s12870-023-04037-w

**Published:** 2023-01-09

**Authors:** Xiaohan Jiang, Kangchen Liu, Huixiang Peng, Jing Fang, Aidi Zhang, Yuepeng Han, Xiujun Zhang

**Affiliations:** 1 Key Laboratory of Plant Germplasm Enhancement and Specialty Agriculture, Wuhan Botanical Garden, Chinese Academy of Sciences, Wuhan, 430074 Hubei China; 2 Center of Economic Botany, Core Botanical Gardens, Wuhan Botanical Garden, Chinese Academy of Sciences, Wuhan, 430074 Hubei China; 3 University of Chinese Academy of Sciences, Beijing, 100049 China

**Keywords:** Peach, Fruit ripening, Organic acid, Gene co-expression network, Dynamic network analysis

## Abstract

**Background:**

Organic acids are important components that determine the fruit flavor of peach (*Prunus persica L. Batsch*). However, the dynamics of organic acid diversity during fruit ripening and the key genes that modulate the organic acids metabolism remain largely unknown in this kind of fruit tree which yield ranks sixth in the world.

**Results:**

In this study, we used 3D transcriptome data containing three dimensions of information, namely time, phenotype and gene expression, from 5 different varieties of peach to construct gene co-expression networks throughout fruit ripening of peach. With the network inferred, the time-ordered network comparative analysis was performed to select high-acid specific gene co-expression network and then clarify the regulatory factors controlling organic acid accumulation. As a result, network modules related to organic acid synthesis and metabolism under high-acid and low-acid comparison conditions were identified for our following research. In addition, we obtained 20 candidate genes as regulatory factors related to organic acid metabolism in peach.

**Conclusions:**

The study provides new insights into the dynamics of organic acid accumulation during fruit ripening, complements the results of classical co-expression network analysis and establishes a foundation for key genes discovery from time-series multiple species transcriptome data.

**Supplementary Information:**

The online version contains supplementary material available at 10.1186/s12870-023-04037-w.

## Background

Peach (*Prunus persica* L. Batsch) belongs to the Prunoideae subfamily of the Rosaceae [1, 2]. As an important economic deciduous fruit, peach fruit ranks sixth in yield in the world [3, 4]. Compared with other perennial fruit crops, peach has a small diploid genome and relatively short juvenile period [5]. It is a good material for studying functional genomes, so it has been considered to be a model species of Rosaceae family [6, 7]. The economic value of peach is mainly determined by quality, taste, aroma, and storage durability [8]. Acid is one of the important factors affecting taste, and its composition and metabolic process are relatively complex. They are affected by genetic factors, physiology, environmental conditions and cultivation measures [9]. Physical location is the first step to isolate and modulate peach acidity during the improvement of peach varieties [10]. The predecessors used fruit acid measurement, amplified fragment length polymorphism (AFLP) markers and bacterial artificial chromosome (BAC) libraries to locate the gene controlling peach acidity at the D site of the fifth chromosome of the peach genome [9, 11–14]. However, due to the variety of organic acids in peach and the complexity of metabolic methods, no specific genes have been found to control the acidity of peach [15].

With the progress of high-throughput biotechnology experiments, large amounts of omics data are available and provide us the chance to investigate the key genes controlling some complex traits by bioinformatics approaches. Among these methods, network analysis is an important tool for characterizing gene relationships with the assumption that network is a common form of complex system [16, 17]. In network science, life system is considered as a molecular network composed of different biochemical reaction pathway modules [18, 19]. With the development of network theory and technology, a lot of different organizational forms of networks in biological system are discovered, such as transcription regulatory network, protein interaction network, metabolism network, and signal transduction pathway. In a biological system, genes and proteins are important components, but what's more important are the relationships between them [20, 21]. Genes that perform similar functions at the same time are more closely related to each other, so we can divide gene sets that perform similar functions through network construction [22, 23]. As one of the most popular methods for network analysis, the weighted gene co-expression network analysis (WGCNA) has been widely used and proved it to be a useful tool for transcript analysis. However, it cannot detect the dynamical mechanism of biological process [24].

The time-series transcriptome data generally contains three dimensions of information, namely time, phenotype and developmental stage, which calls 3D transcriptome data [25]. It can provide the information about the stages of development for detecting the dynamic mechanism [26, 27]. The transcriptomes of different varieties with huge phenotypic differences can reveal the genes that exhibit variety-specific differential expression. As a time-ordered (TO) gene co-expression network analysis (GCN) tool for dealing with this kind of 3D data, TO-GCN method can reveal the dynamics of gene expression and the transition of biological processes [28, 29]. Specifically, TO-GCN not only avoids time-point alignment, but also overcomes the trouble from normalization between conditions. It will not lose time information because the average value represents the level of each time series. In addition, it will not generate new gene clusters that do not belong to the original data due to the fusion of multiple datasets after normalization. Different from classical co-expression network analysis, it considers continuous time points in transcriptome data. This method has been validated by many experimental methods [28].

In this study, we used 3D transcriptome data from 5 different varieties of peach to construct gene co-expression networks throughout fruit ripening. With the network inferred, the time-ordered network comparative analysis was performed to select high-acid specific gene co-expression network and then clarify the regulatory factors controlling organic acid accumulation. The difference of synthesis and metabolism of different organic acid components in high-acid varieties and low-acid varieties results in the total acid content. The time delay for the respective synthesis and degradation of organic acid components was considered for the comparative analysis of different developmental stages. After the co-expression patterns under high acidity and low acidity were compared, we obtained 20 candidate genes related to organic acid metabolism in peach. Overall, our study provides new insights into the dynamics of organic acid accumulation during fruit ripening and establishes a foundation for key genes discovery from time-series transcriptome data of multiple species.

## Results

### Phenotypic diversity of organic acids during fruit development

**Fig. 1:**
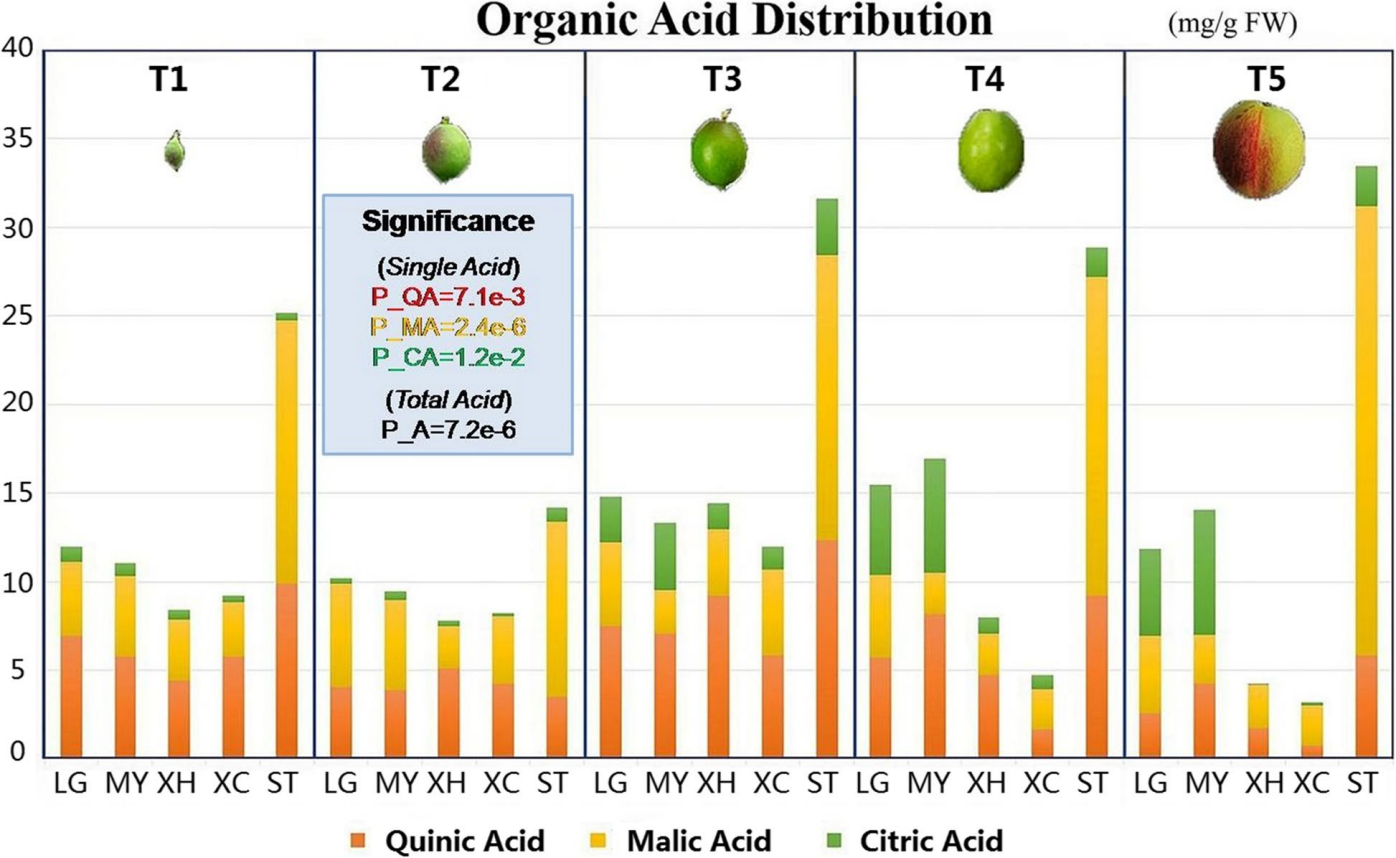
Distribution and dynamics of three organic acids of five peach varieties in five developmental stages. LG stands for Legrand, stands MY for Meiguowanyou, XC stands for Xiacui, XH stands for Xiahui and ST stands for Shantao. T1 to T5 represent 5 developmental stages, corresponding to 5 sampling time points. The unit of different organic acid content of each peach variety measured by HPLC is micrograms per gram)

Sampling is divided into five periods, i.e. the young fruit period, the first expansion period, the hard core period, the second expansion period and the maturity period (Fig. 1). In order to maintain a consistent time sequence and facilitate the operation of the sampling personnel, sampling was carried out on 34, 50, 75, 97 and 117 days after full bloom (DAF), respectively [30–32]. The samples were sampled from three biological replicates, and the five peach varieties were basically at similar stages of development. The stages of fruit development are divided into five stages, i.e. T1-T5. T1 (34 DAF) corresponds to the young fruit stage when the ovary begins to develop. T2 (50 DAF) corresponds to the first expansion stage and the fruit expands rapidly at this time. T3 (75 DAF) corresponds to the hard core stage and at this time development is delayed and the core hardening. T4 (97 DAF) corresponds to the second expansion period and at this time the fruit expands again. T5 (117 DAF) corresponds to the mature period and the fruit basically stops growing and matures gradually.

The composition of peach organic acids is analyzed from two dimensions, one is the total amount of organic acids, and the other is the proportion of organic acids. These two points are the key factors affecting the economic value of peaches. The total amount of organic acids determines the sugar-acid ratio of fruit, and the proportions of malic acid and citric acid are also the important factors that affect the peach flavor. In terms of acid content, Shantao (ST) is the highest one in total acid content. The high acidity is mainly related to the genetic background of the wild species when the factors of different fruit sizes are excluded. The organic acids in ST are mainly composed of malic acid and quinic acid, while citric acid has little effect on the total acid and its content fluctuates slightly from T1 to T5. After the hard core period (T3), the content of quinic acid decreases slowly while malic acid continues to accumulate from the first expansion period (T1). The above two points cause malic acid to account for the largest proportion of acid content of ST in the maturity stage (T5).

The total acid content of the two nectarine varieties is almost always higher than that of the two hairy peach varieties. The acid content difference between the nectarine varieties and hairy peach varieties in the first three periods is small, but the difference in the latter two periods is huge. Two nectarine varieties Legrand (LG) and Meiguowanyou (MY) are high-acid varieties, and two hairy varieties Xiacui (XC) and Xiahui (XH) are low-acid varieties. From Fig. 1, we can find that this difference is not only caused by the accumulation or degradation of a certain acid, but also has a huge relationship with the simultaneous change of three organic acids. In the first two periods, malic acid and quinic acid accounted for a larger proportion of the total acid content of high-acid varieties. From the hard core period (T3), citric acid accumulates rapidly until the mature period (T3), accounting for 1/3 to 1/2 of the total acid. The total acid content of low-acid varieties was mainly dominated by quinic acid in the first three periods, and the proportion of citric acid in it was always relatively small and stable. Since the degradation rate of quinic acid in the later period is higher than that of high-acid varieties, the degradation rate of malic acid is slower than that of quinic acid. Therefore, the total acid content was gradually converted to be dominated by malic acid from the T3 period. It can be observed from the line chart that the changing trends of the three organic acids in different varieties have their own characteristics. The main changing trend of malic acid in the four cultivars is declining, while the changing trend in the wild species is exactly the opposite. Citric acid showed an overall upward trend in high-acid varieties. It rose slightly and then slowly declined in low-acid varieties, but it changed more irregularly in wild species. These two points show that wild germplasm resources and cultivars are very different in certain traits. However, the quinic acid of these five selected varieties are all degraded at the later stage of development, and their changing trends are relatively consistent. Therefore, there are similarities between wild species and cultivated species.

### Spatiotemporal dynamics of gene expression during fruit development

In order to investigate the dynamic changes of gene expression in fruit development, we conducted RNA-seq experiments on total RNA extracted from five stages of fruit development from the five peach varieties, LG, MY, XC, XH, and ST. Through three independent biological replicates of each variety, a total of 3.62 billion high-quality reads were obtained. By using Hisat2 tool, the reads with an average of 48 million reads per sample and an average Q30 of 89% were mapped to the peach genome and resulted in the average comparison rate 92%. Then, by using Stringtie tool combined with the annotation file, a total of 26873 known peach genes were identified. The unique alignment reads (39-48M) of each sample continue to be processed using Stringtie components to determine the standardized expression abundance, i.e. fragments per kilobase transcribed length per million marker reads (FPKM) per transcription.

Overall, approximately 92.6% of genes are expressed in at least one out of 25 samples. There are certain differences in the number of expressed genes in different periods and different varieties. LG and MY expressed the least genes in the T5 period (75.8% and 76.4%, respectively) and the most expressed genes in the T1 period (79.2% and 79.8%, respectively). Different from the two nectarine varieties, the period in which the other three wild peach varieties expressed the least number of genes was in the T3 period, which was 76.2%, 76.1%, and 76.9%, respectively. The same was that the period in which they expressed the highest number of genes was also in T1 period. During the period, the proportion of expressed genes was 79.7%, 81.7% and 81.8%, respectively.

**Fig. 2:**
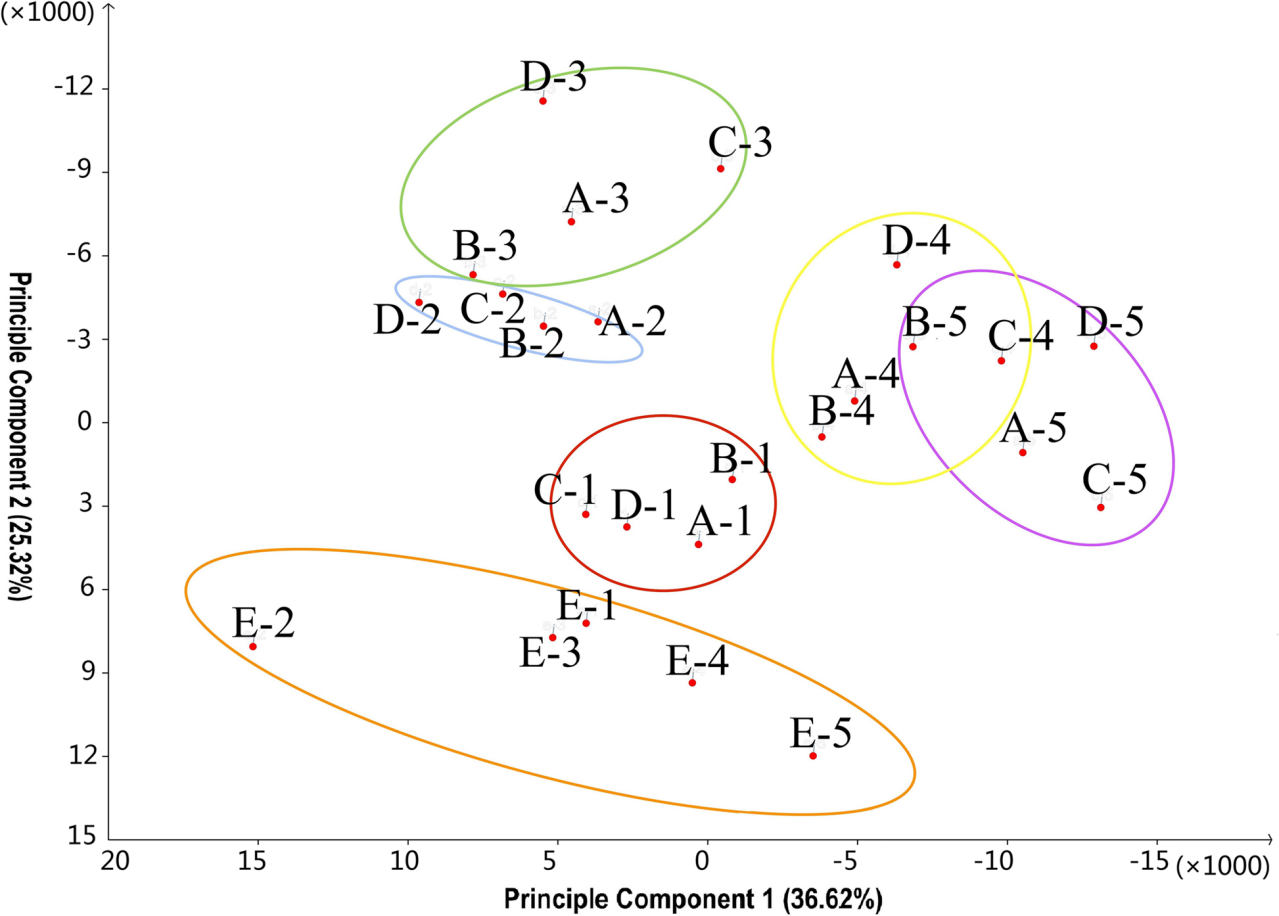
Principle Component Analysis. Letters a - e represent Legrand(LG), Meiguowanyou(MY), Xiacui (XC) and Xiahui (XH) and , Shantao (ST), 1-5 represent five stages of peach fruit development. The explanation rate of population variance of Principal component 1 is 25.32%,the explanation rate of population variance of Principal component 2 is 36.62%. The ellipses of different colors divide the samples of different periods into different categories

In next step, the obtained FPKM matrix (Supplementary Table S1) was used for principal component analysis [33]. To facilitate the display the result with the large number of samples, we calculated FPKM value of each biological repeat after the arithmetic average and then conducted principal component analysis. From the obtained scatter plot (Fig. 2), we found that the overall distribution of the ST variety in the first quadrant was separated from other samples, indicating that the gene expression pattern of this variety was different from other varieties, but from the view of the *x* projected from PC1 on the axis, the period position of ST can be matched with different developmental stages of other varieties. This conclusion verifies the similarity and the difference of the phenotypes in the previous conclusion from the genetic level. The similarity of the four cultivars in time was very high. Samples of different varieties in the same period can be grouped together respectively, which was much higher than the similarity of samples of the same variety in different periods. This shows that the genes of different peach varieties have similar expression patterns in the same development period. The red ellipse is the first period, the blue the second period, the green the third period, the yellow the fourth period, and the purple the fifth period. We can observe that the sample expression patterns of T2 is similar to that of T3 while the sample expression patterns of T4 is similar to that of T5, which is consistent with the actual fruit development.

### Network module related to fruit development and acidity accumulation

**Fig. 3:**
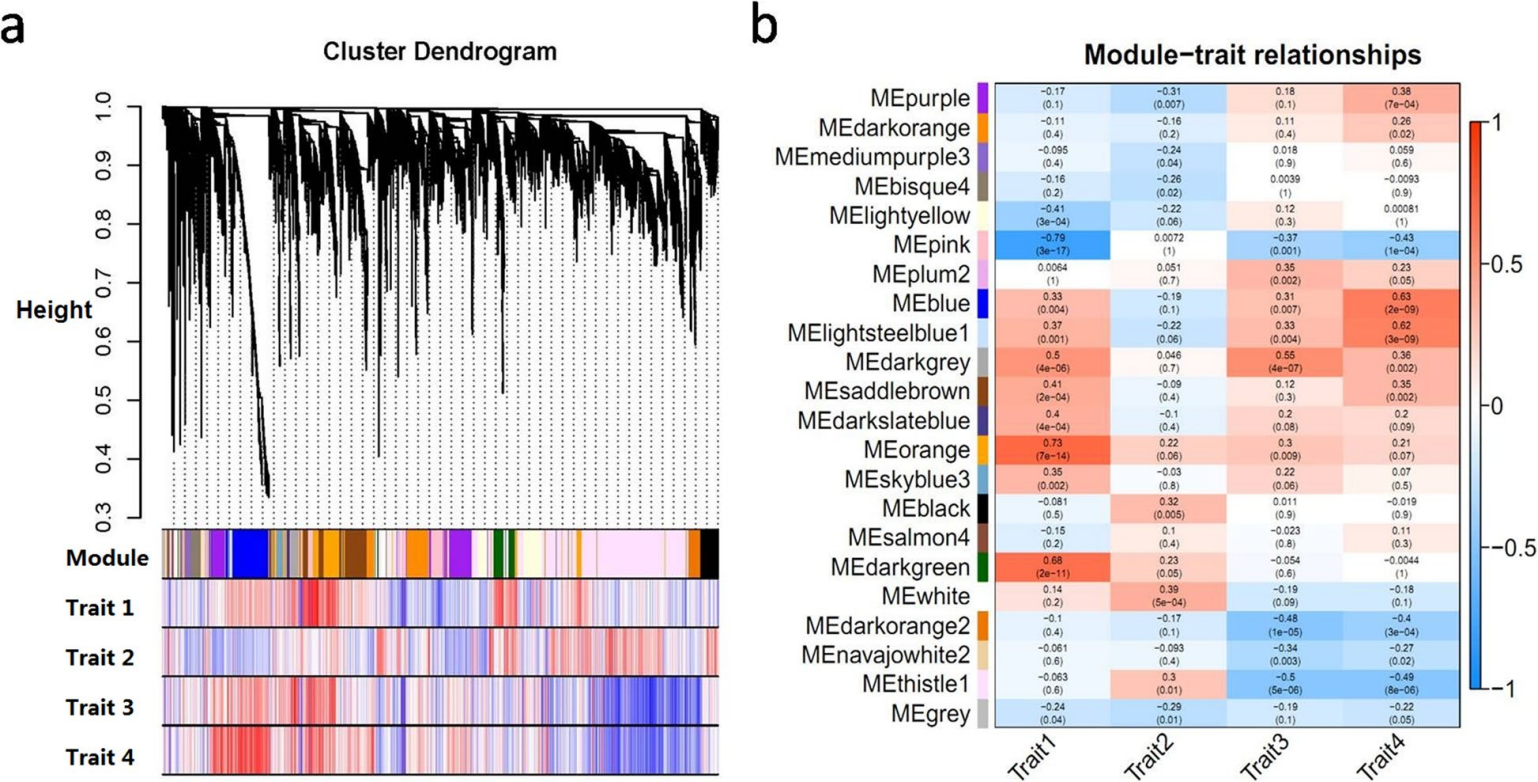
**a**. Hierarchical clustering tree (dendrogram) of genes based on coexpression network analysis in four cultivars. Each ‘leaf’ (short vertical line) corresponds to individual gene. The genes were clustered on the basis of dissimilarity measure (1-TOM). The branches correspond to modules of highly inter- connected genes. The color rows below the dendrograms indicate module membership in four cultivar and their corresponding modules in different trait. **b**. Module-sample feature correlation analysis

The weighted gene co-expression network was analyzed using the gene expression matrix data. By calculating the correlation coefficient between each gene pair, different gene modules were obtained by hierarchical clustering based on the weighted correlation, and then the gene modules related to the traits were identified according to the correlation with the four phenotypes (Fig. 3a). As a result, a total of 22 modules correlated with traits were identified. Among them, the orange module and the dark green module matched with malic acid were positively correlated, while the pink module and the light yellow module were negatively correlated. The white module and the black module matched with citric were positively correlated, while the purple module was positively correlated. And the dark grey module and the plum2 module matched with quinic acid were positively correlated, while the thistle module and the darkorange2 module were negatively correlated. All *P*-values were less than 0.05, indicating that the result of the classification is significant (Fig. 3b). We obtained 950 possible genes related to malate metabolism, 626 possible genes related to citric acid metabolism, and 1574 possible genes related to quinic acid metabolism from the modules that were closely related to traits.

**Fig. 4:**
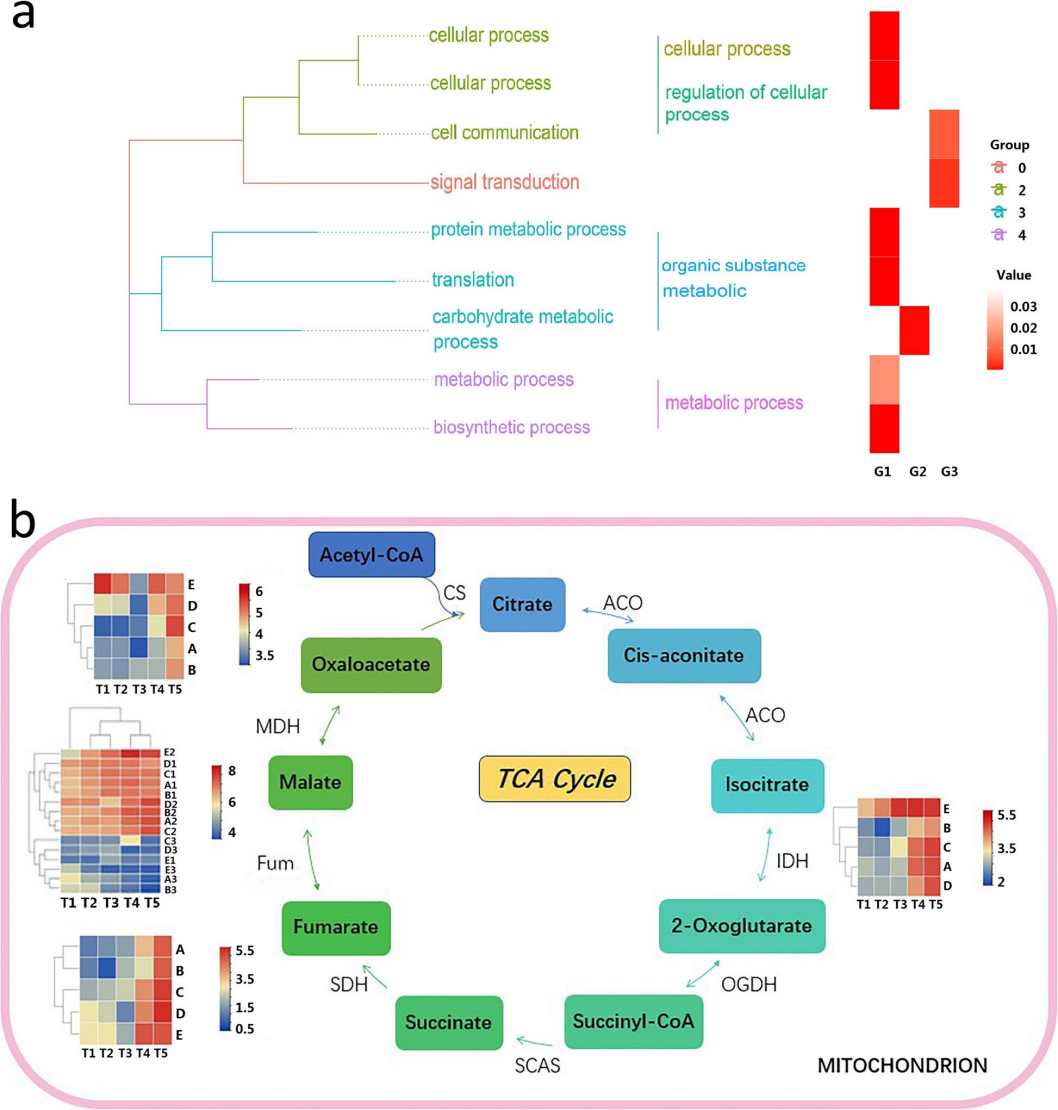
Functional clustering analysis**. a**. GO cluster of genes in acid-related modules. **b**. Expression of genes in acid-related modules taking part in TCA cycle

Selecting the simplified plant Gene Ontology (GO) set as the enriched background, we performed gene ontology functional enrichment for the genes in each gene module and only the significant results were retained. According to the classification in GO, the BP (Biological Process), MF (Molecular Function), and CC (Cellular Component) lists related to each trait are respectively summed to do GO clue synthesis. To obtain more accurate gene set, the related metabolic pathways compared with the result of differentially expressed genes (DEGs) were annotated. We selected the module with the largest correlation with malic acid as a demonstration (Fig. 4a). Both positive and negative correlation modules were included. After performing GO clustering on the genes, the entries with a *p*-value less than 0.05 were de-redundant. We can find that the functions of the modules related to this trait were mainly focused on organic substance metabolism, cellular process and signal transduction. The result showed that accumulation and degradation of malate involved a large number of organic substances in different cellular processes including the tricarboxylic acid (TCA) cycle in mitochondria (Fig. 4b). The activity of proton pump on the vacuole was also involved in a large number of signal transduction. The functional clustering of genes in malate-related module obtained by WGCNA was consistent, showing that the results are scientifically supported.

### Candidate genes for organic acid accumulation during fruit development

**Fig. 5:**
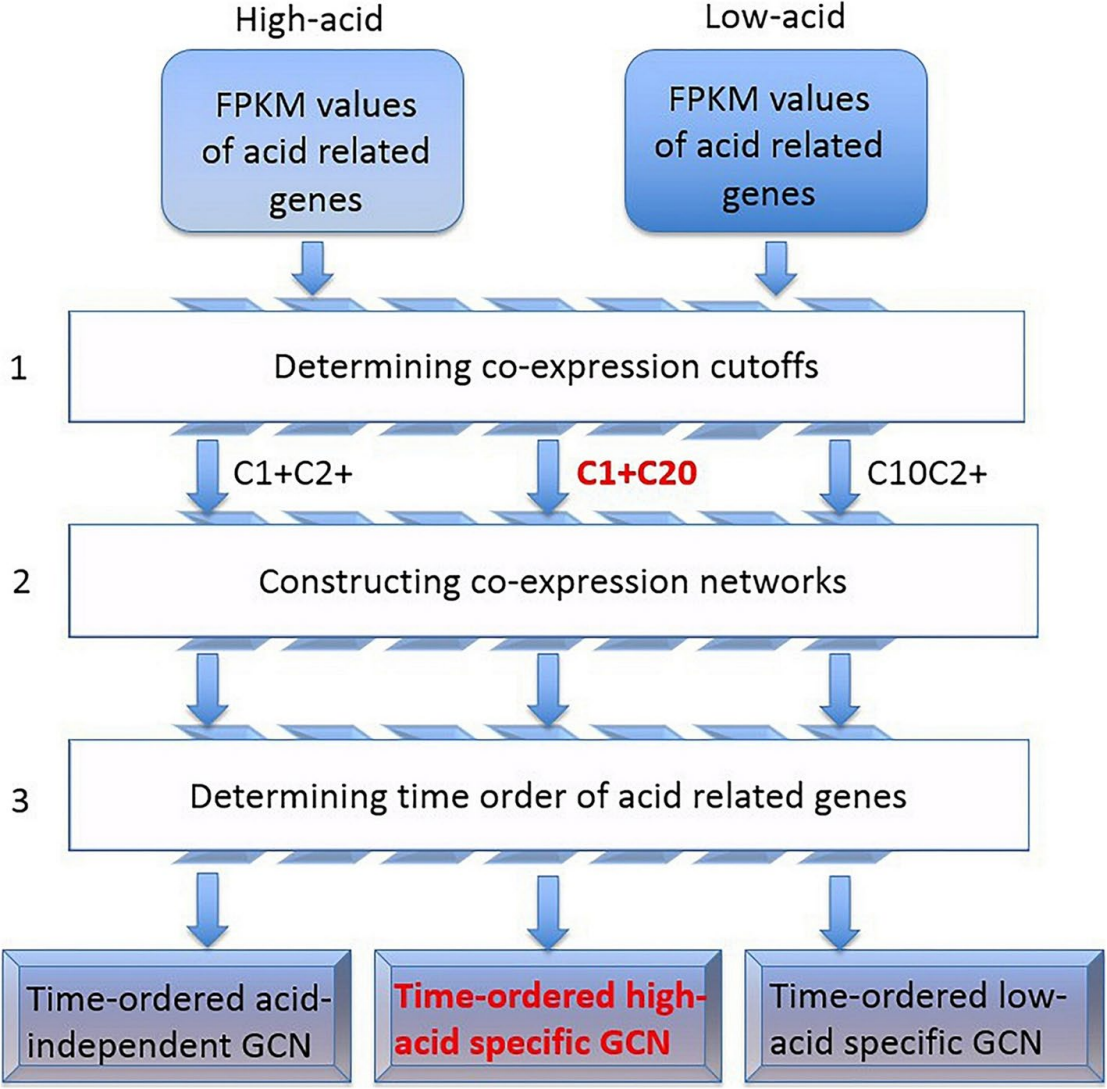
Three steps to construct an acid-related time-series gene co-expression network. The independent of acid (C1+C2+), high acid specific (C1+C20) and low acid specific (C10C2+) GCNs are shown as three examples. Lable +/-represent positive/negative co-expression

**Fig. 6.**
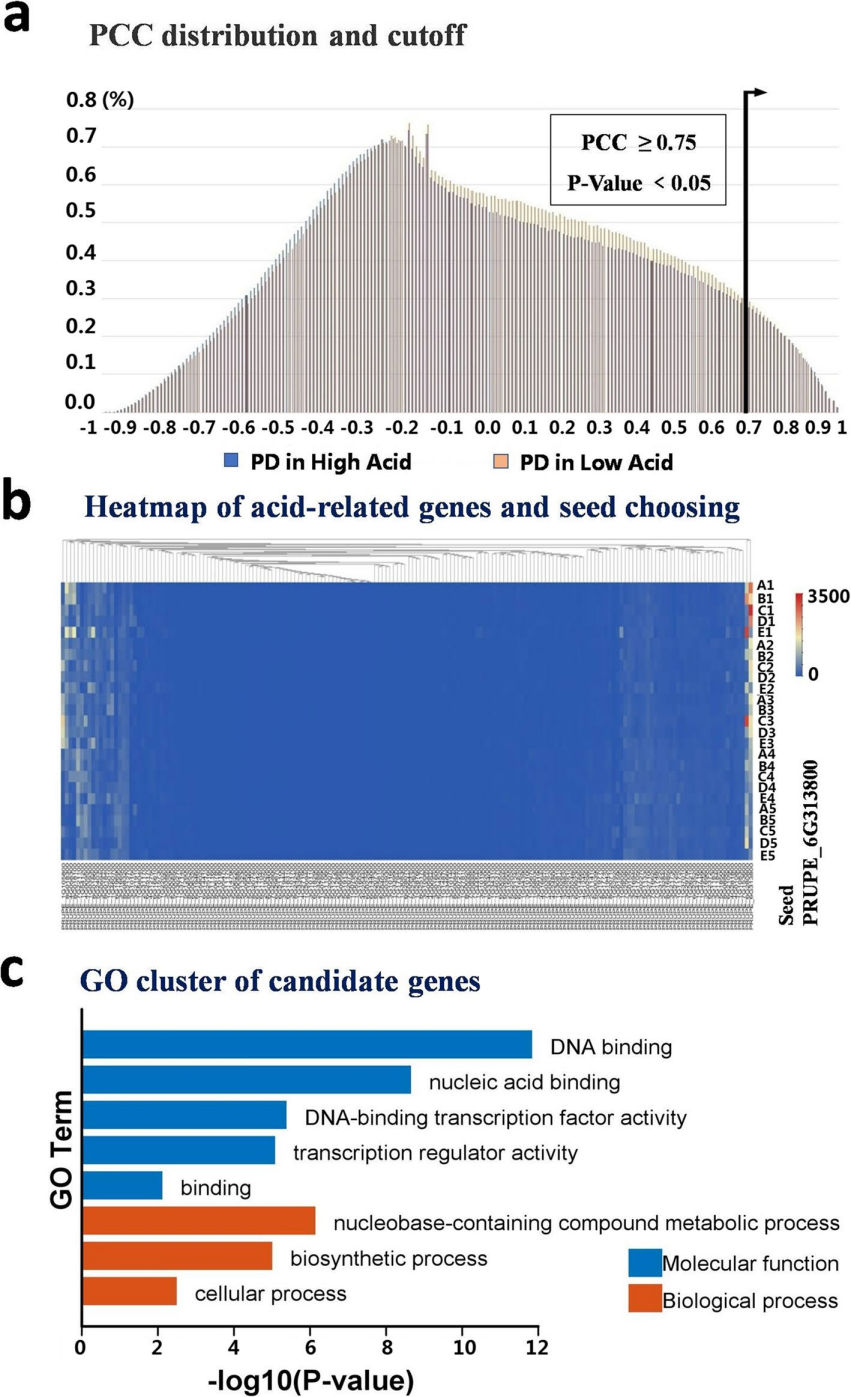
**a**. The distributions of PCC values between TF genes over the time-series FPKM values under LD (blue bars) and under TD (red bars). The cutoff is set to 0.75 with *p* < 0.05. **b**. Heatmap of acid-related genes for choosing a seed in step 3. Each column represents a gene, each row represents a developmental stage, and each crossed color block represents the expression level of a gene in that period. **c**. GO cluster of 20 candidate genes related to organic acid metabolism

TO analyze the accumulation of organic acids in fruits of high-acid and low-acid varieties, the time-ordered gene co-expression network analysis (TO-GCNs) was implemented on the peach transcriptome with 5 developmental stages (Fig. 5). Firstly, all the transcription factors (TFs) and genes on the acid-related pathway in the GDR (https://www.rosaceae.org/) annotation were collected for constructing the gene co-expression network which directly or indirectly affects the accumulation and degradation of peach organic acids in different developmental stages. The network included the genes involved in TCA cycle and the related proton pump genes on the vacuole, etc. With FPKM values, the *Pearson* correlation coefficients (PCCs) of 182 acid-related gene pairs and all transcription factors under high-acid varieties and low-acid were calculated separately (Supplementary Table S2). Then, the PCC values obtained were used to generate the distribution matrix of probability density function (PDF) and cumulative density function (CDF). According to the CDF, we obtained positive and negative reference cutoffs under each condition when the *p*-value is less than 0.05, which means the result is significant (Fig. 6a).

As a statistical result, the significant (*P*-value <0.05) positive and negative cutoffs for PCC values are 0.75 and -0.60. If PCC>0.75, two genes are defined as positive co-expressed (indicating as C1+ or C2+). In addition, if -0.5≤PCC<0.5, two genes are defined as not co-expressed (indicating as C10 or C20). If PCC < -0.60, it means negative expressed (indicating as C1- or C2-). We obtained 8 time-series gene co-expression networks (TO-GCNs), which were used to predict the time node order of genes in GCN. We jointly considered the co-expression status under C1 and C2. If both genes are positively co-expressed in high-acid varieties and low-acid varieties, the two genes belong to the C1+C2+ relationship set. Similarly, two genes belong to the C1+C20 relationship set (Supplementary Table S3) if they are only expressed in high-acid varieties but not co-expressed in high-acid or low-acid varieties. We focused on the C1+C20 relationship which may include key genes involved in the metabolism of organic acids in high acid varieties. Other group may be discussed in other follow-up studies.

The expression levels of organic acid metabolism-related genes in different periods were presented in the form of heatmap (as shown in Fig. 6b), and the first gene to be expressed was selected as the seed of predict the sequence of time nodes of the genes in GCN. From the statistical table, we can find that the genes expressed in these five developmental stages have been redistributed into 8 time points. With the help of combination with the annotated function corresponding to each gene, we can find that organic acids accumulate firstly and then degrade, which matches the actual biological process. The time period of redistribution is reasonable, so we can infer that the genes with greater connectivity within these selected genes have a certain effect on accumulation and degradation of organic acids. From the total PCC table, the genes with a high degree of association make up the small network component genes were selected as candidate genes. Our previous study has verified that one of them, PRUPE.5G008400, is related to citric acid metabolism [34]. We used cytohubba [35], a plugin of the Cytoscape_v3.9.0 [36], to select the hub genes from this gene set. In the result, some genes can be discovered as candidate genes. After performing GO clustering on the candidate genes related to organic acid metabolism, we can find that the functions of these genes were mainly focused on biosynthetic process, cellular process and DNA binding (Fig. 6c). Even though the number of genes used for clustering is reduced, their functions are similar to the results of malic acid-related module clustering mentioned above (Fig. 4a), which indicates that this method enables us to screen candidate genes more accurately without changing the direction of target traits to a certain extent.

As a result, we obtained 20 candidate genes related to organic acid metabolism in peaches (Table 1). We can find that no matter what is the permutation or combination, after selecting the first expressed gene as the seed gene, the action phase of two genes after sequencing are very close, further demonstrating that it is feasible to use this method to find functionally related genes from the vicinity of known key genes. In a word, our analysis was an effective way to identify candidate genes related to specific metabolic pathway.

**Table 1:** Candidate genes related to organic acid metabolism

Rank	Name	Count	Annotation
1	PRUPE_3G046900	7	Myb-like DNA-binding domain
2	PRUPE_1G274300	6	No apical meristem (NAM) protein/regulation of transcription, DNA-dependent
3	PRUPE_3G271200	6	Sequence-specific DNA binding transcription factor activity
4	PRUPE_6G112000	6	No apical meristem (NAM) protein/regulation of transcription, DNA-dependent
5	PRUPE_3G084600	9	AP2 domain/sequence-specific DNA binding transcription factor activity
6	PRUPE_3G198700	12	Protein binding
7	PRUPE_6G280800	2	Magnesium ion binding/pyruvate decarboxylas
8	PRUPE_1G390000	5	No apical meristem (NAM) protein
9	PRUPE_6G303900	6	Protein dimerization activity/MADS box transcription factor
10	PRUPE_6G121700	12	SRF-type transcription factor (DNA-binding and dimerisation domain)
11	PRUPE_1G339800	5	Malate dehydrogenase, NAD binding domain
12	PRUPE_6G004400	3	Sequence-specific DNA binding transcription factor activity/EREBP-like factor/SHN (SHINE), DNA BINDING
13	PRUPE_7G066700	10	AP2 domain/sequence-specific DNA binding transcription factor activity
14	PRUPE_1G538700	6	Protein dimerization activity/SRF-type transcription factor (DNA-binding and dimerisation domain)
15	PRUPE_4G132100	5	GRAS domain family
16	PRUPE_8G166900	6	bHLH-MYC and R2R3-MYB transcription factors N-terminal/protein dimerization activity
17	PRUPE_1G441700	14	Myb-like DNA-binding domain
18	PRUPE_2G278500	8	Domain of unknown function (DUF313)
19	PRUPE_1G037800	4	AP2 domain/ sequence-specific DNA binding transcription factor activity
20	PRUPE_7G206900	4	Heat shock transcription factor/nucleus/response to heat

## Discussion

This article provides new insights into the dynamics of organic acid accumulation during peach fruit ripening, complements the results of classical co-expression network analysis. As we all know that peach has a relatively small genome (~230 Mb/haploid) and a short juvenile phase (2–3 years), which makes it suitable to serve as a model for investigation of the inheritance mechanism of fruit quality [37]. The growth and ripening of fleshy fruit is typically accompanied by numerous biochemical and physiological changes, such as increase of soluble sugars or decline of acidity [38]. Organic acids that are important metabolites of the tricarboxylic acid cycle occupy a central position in plant metabolism. However, the major gene influence the accumulation of acidity is still unknown. Current genome researches have strengthened the genetic basis underlying these two internal quality properties for fruit flavor improvement in many fruit crops [39–43].

The Chinese peaches are regarded as the most influential germplasm in the history of global peach breeding [44, 45]. There are different selections between eastern and western peach breeding programs, two typical flavor types: sweet, low-acid vs. sweet, acid taste, respectively favored by eastern and western consumer [37, 46]. This situation provides us a chance to select stable varieties with wide acidity differences to find the genes which influence the accumulation of organic acid in peach. In most ripe fruits, malate and citrate are the predominant organic acids [47]. Fruit acidity depends on both the content and composition of organic acids [48] Peach fruits contain mainly three kinds of organic acids, citrate, malate and quinate [49]. Such great differences in both malate and citrate contents along with a relatively small difference in quinate content were also noted in a recent study [50, 51]. A number of studies have shown that peach fruit acidity is mainly controlled by the D locus on chromosome 5 (Chr5), with low acidity being dominant over high acidity [52, 53]. Interestingly, apart from its role in controlling fruit acidity, the D locus may also have a minor effect on fruit sugar accumulation [54–56], which give us new thought to comprehend the change of organic acid content.

The pursuit of high fruit quality is always the aim of the researchers in fruit science. With the development and innovation of technology, mining key genes involved in the metabolic pathway of organic acids is becoming possible. From the earliest use of molecular markers to construct genetic maps and large-scale genome-wide association analysis (GWAS) for quantitative trait loci (QTL) mapping to the use of genomic structural variation to correlate traits, the study of organic acids in peach has made much progress. But the major genes that control the accumulation of organic acids in peach have not yet been determined. According to the sampling characteristics of RNA-seq data, this study introduced a specific network method to infer their biological functions in the living environment based on the interactions between genes, and tried to identify some candidate genes missed by other methods. It provided new insight for further analyzing the mechanism of organic acid accumulation in peach.

## Conclusions

According to the peach genome annotation and previous literature reports, we retrieved and filtered genes that are closely related to acid metabolism, and analyzed the corresponding transcriptome data as a supplement. We carried out expression analysis, and looked for the intrinsic relationship through the expression of these genes at different stages of fruit development. First, we construct a co-expression network based on the above transcriptome data. Then screen out the network modules related to organic acid synthesis and metabolism under the comparison conditions of high acid and low acid. Finally, hub gene was selected as candidate gene by network analysis. It is worth mentioning that we used a new method called TO-GCN method to obtain candidate genes, which is especially designed for 3D data that including time, phenotype and gene expression. Compared with the classical co-expression network analysis method [57], we added the consideration of time series. That is, when constructing a co-expression network, the dynamic changes of the network are considered, and a network with more connections is constructed through the comparison of phenotypes, thereby mining genes that cannot be captured by traditional methods.

## Methods

### Plant materials and sampling

All peach cultivars used in the study are grown at Northwest A&F University, Yangling, China. Five varieties including Legrand (LG) and Meiguowanyou (MY) (high-acid varieties), Xiacui (XC) and Xiahui (XH) (low-acid varieties) and Shantao (ST) (wild variety) were involved in study. A total of 75 samples for five time points and three random biological repetitions were selected for RNA sequencing (RNA-Seq). Five time points include young fruit period (T1), first expansion period (T2), hard-core period (T3), second expansion period (T4) and maturity period (T5). The fruits used were randomly collected in our study to ensure the consistency of sample collection, and each fruit is taken from the middle of the tree and try to ensure the same position. Fruit samples were collected in 2017 and each variety had at least ten fruits. After the fruit samples were pitted, the flesh was cut into small pieces and immediately frozen in liquid nitrogen, then stored at −75°C for sequencing and further experimental analysis.

Previous studies have shown that nectarine grows larger and larger during fruit development. MY and LG are nectarine varieties, and their fruits are still growing in the T3 period [57]. But their changes in the development period are basically the same as that of hairy peaches. ST is a wild variety of peach. Its change in size from T1 to T5 is not obvious compared to other four cultivars. However, from the perspective of the accumulation of acidity, its growth stage is basically the same as other four varieties. The sampling time points inferred from above are very representative and can basically distinguish the stages of peach development. Each sample can reflect the status of the transcription products of the peach variety at the current development stage. In addition, we selected three biological repetitions instead of technical repetitions to mutually verify the unity of developmental periods. Due to the difference of varieties, even under the same cultivation environment, the development period of all samples cannot be completely consistent. We used biological repetition to ensure the consistency of samples of a single variety in a single period, thereby enhancing the representativeness of the transcriptome of the sample performance and improving the stability and reliability of transcriptome data.

### Measurement of three organic acids

According to our previous reports [58], the HPLC was used to test the main organic acid content in five different varieties of peach fruits. The mesocarp of peach fruits from each replicate was ground into powder in liquid nitrogen using an A11 basic Analytical mill (IKA, Germany). Approximately 0.5 g of powder was dissolved in 6 mL deionized water obtained from a Milli-Q Element water purification system (Millipore, Bed ford, MA, USA). The mixture was extracted in an ultrasonic bath for 15 min, and then centrifuged at 5000×g for 15 min. The supernatant was purified using a SEPC18 syringe (Supelclean ENVI C18 SPE), and filtered through a 0.22 μm Sep-Pak filter (ANPEL, China) to 2 mL clean centrifuge tube, stored at −4 °C for test. The filtered supernatants were transferred to sutosampler vials (CNW, 9 mm), and put into the Agilent 1100 Series autosampler to measure organic acid content using an Agilent 1260 Infinity HPLC system (Milford, MA, USA). Chromatographic separation was conducted with an Athena C18-WP column (CNW, 4.6 × 250 mm, 5 μm), and the column temperature was maintained at 40 °C. The mobile phase was 0.02 mol/L KH2PO4 solution with a pH value of 2.4. The elution was performed at the flow rate of 0.8 mL/min. The acid concentration was quantified using ultraviolet (UV) absorbance detection at 210 nm. Three technical replicates were performed for each sample and three biological replicates for each treatment. The acid concentration was calculated by comparison with the values obtained from a standard curve, and expressed in mg/g fresh weight (FW).

### RNA-seq data arrangement

Raw data were trimmed using Trim Galore with Q > 30 [59] and then mapped to the reference genome of peach using HISAT2 and Stringtie [60–62]. The number of reads mapped to each gene was calculated using the HTSeq v0.6.0 software [63]. The gene expression levels was estimated based on the value of expected number of fragments per kilo-base of transcript sequence per millions base pairs sequenced (FPKM).

### Gene co-expression network analysis

Weighted gene co-expression network analysis (WGCNA) is a systematic biological method used to describe gene association patterns between different samples. It can be used to identify highly synergistically changing gene sets [24]. Candidate genes were identified based on the connectivity of the gene set and the association between gene set and phenotype. Firstly, the *Pearson* correlation coefficients (PCCs) between genes were calculated based on gene expression data and then been used to measure the weighted co-expression network. Secondly, the gene modules related to different kind of organic acids were identified. Based on the weighted correlations, the hierarchical cluster analysis was performed, and the clustering results according to the set criteria were divided to obtain different gene modules which were represented by the branches of the cluster tree and different colors. Thirdly, the correlation between the genetic module and the phenotype was calculated to identify the modules related to the trait using the phenotype information. Fourthly, the interactions among different modules were constructed to identify the driver genes of interest from the key modules and predict the function of some genes. Lastly, the topological overlap (TOM) matrix was exported to visualize the graphs using R packages.

### GO and pathway enrichment analysis

The gene ontology enrichment analysis for different module sets was performed using TBtools [64] and clusterProfiler [65]. Firstly, the species-related annotation package was obtained from AnnotationHub (https://bioconductor.org/packages/release/bioc/html/AnnotationHub.html). Secondly, the sub-database was constructed according to the concept of gene ontology for following analysis. Thirdly, we selected GO entries with *P*-value less than 0.05 to draw the phylogenetic tree. Lastly, the de-redundant GO entries obtained with evolutionary relationships were used for annotation.

### Dynamic network analysis

To start the analysis in the workflow, two lists of FPKM values for the whole genes and specific target genes at different sample points under two conditions were generated. The dataset used in this work is named 3-D data for different species, trait and developmental stages. Three programs are named as Cutoff, GCN, and TO-GCN. We used C++ source code (.cpp) and compile them into executable files. Firstly, we calculated PCC values for each TF gene pair under each condition. With PCC values obtained, we generated a distribution of probability density function (PDF) and cumulative density function (CDF). According to the CDF, we can suggest positive and negative cut-off values for each condition, *p*<0.05. Secondly, we constructed eight GCN co-expression types under two conditions (C1 and C2): C1+C2+, C1+C20, C1+C2-, C10C2+, C1-C2+, C1-C2-, C1-C20, and C10C2-, where +, -, 0 indicate positive, negative, and no co-expression, respectively. The output file for each GCN was listed in a comma-separated value (.csv) format. These five columns represent the acidity gene ID, co-expression type, gene ID, PCC under condition 1, and PCC under condition 2. Besides four parameters in the previous step, four more parameters were provided to indicate the positive cut-off values of conditions 1 and 2 and the negative cut-off values of conditions 1 and 2. Lastly, we determined the chronological order of the nodes in GCN. The time sequence was assigned by the breadth first search (BFS) algorithm which starts with a selected set of seed nodes. We used positive cut-off values for conditions 1 and 2. Two other parameters were used to indicate the seed node gene ID and co-expression type. For co-expression type, the numbers 0, 1, and 2 represent C1+C2+, C1+C20 and C10C2+, respectively.

## Supplementary Information

**Additional file 1: Table S1.** Gene expression data (FPKMs) used for principal component analysis.

**Additional file 2: Table S2.** Pearson correlation coefficients (PCCs) of acid-related gene pairs and all transcription factors.

**Additional file 3: Table S3.** Pearson correlation coefficients (PCCs) of genes in C1+C20 gene set.

## Data Availability

The datasets analysed in this study are available in the NCBI SRA database, https://www.ncbi.nlm.nih.gov/sra/PRJNA626460 and https://www.ncbi.nlm.nih.gov/sra/PRJNA787711 (accession BioProject number: PRJNA626460/PRJNA787711). The data supporting the findings of this study are available at https://github.com/zhanglab-wbgcas/PFR.

## Abbreviations

AFLP: Amplified fragment length polymorphism
BAC: Bacterial artificial chromosome
WGCNA: Weighted gene co-expression network analysis
TO-GCN: Time-ordered gene co-expression network analysis
ST: Shantao
MY: Meiguowanyou
LG: Legrand
XC: Xiacui
XH: Xiahui
FPKM: Fragments per kilobase transcribed length per million marker reads
GO: Gene Ontology
BP: Biological process
MF: Molecular function
CC: Cellular component
DEG: Differentially expressed gene
TCA: Tricarboxylic acid
TF: Transcription factor
PCC: *Pearson* correlation coefficient
PDF: Probability density function
CDF: Cumulative density function
HPLC: High Performance Liquid Chromatography
GWAS: Genome-wide association analysis
QTL: Quantitative trait loci
